# Supplementary material for: Increased Risks of Death and Hospitalization in Influenza/Pneumonia and Sepsis for Individuals Affected by Psychotic Disorders, Bipolar Disorders, and Single Manic Episodes: A Retrospective Cross-Sectional Study
Source: J Clin Med. 2021 Sep 26;10(19):4411. doi: 10.3390/jcm10194411 (PMC8509221; doi:10.3390/jcm10194411)
Supplement: Supplementary file 1 [file jcm-10-04411-s001.zip › jcm-1253773-supplementary.pdf]

Supplementary Table S1: Deaths associated with influenza/pneumonia between 1 January 2018 and 31 December 2019 in patients with severe mental disorder vs reference population according to age and comorbidities.

Age Group [Years]	According to comorbidity [ab]	Population with severe mental disorder [c]			Reference Population			OR (95% CI)	P value
		Population size	Deaths	%	Population size	Deaths	%		
Across all Age groups	**Total**	97,034	439	0.45	7,683,693	16,902	0.22	2.06 (1.87-2.27)	<0.001
	Diabetes	7,467	124	1.66	315,521	3,137	0.99	1.68 (1.40-2.02)	<0.001
	Cardiovascular disease	6,780	136	2.01	560,754	8,232	1.47	1.37 (1.16-1.63)	<0.001
	Hypertension	10,048	129	1.28	769,321	8,085	1.05	1.22 (1.03-1.46)	0.023
	Chronic lung disease	5,335	79	1.48	234,227	2,774	1.18	1.25 (1.00-1.57)	0.049
	None of the above	77,302	169	0.22	6,474,647	5,285	0.08	2.68 (2.30-3.13)	<0.001
20-39	**Total**	28,725	5	0.02	2,612,883	111	0.00	4.1 (1.67-10.04)	0.002
	Diabetes	544	X[d]	-	23,012	X	-	-	-
	Cardiovascular disease	147	0	0.00	6,528	5	0.08	-	-
	Hypertension	283	0	0.00	10,258	4	0.04	-	-
	Chronic lung disease	989	0	0.00	41,988	9	0.02	-	-
	None of the above	26,929	4	0.01	2,535,282	91	0.00	4.14 (1.52-11.27)	0.005
40-59	**Total**	38,813	53	0.14	2,529,453	553	0.02	6.25 (4.72-8.29)	<0.001
	Diabetes	2,640	20	0.76	61,004	76	0.12	6.12 (3.73-10.03)	<0.001
	Cardiovascular disease	1,408	9	0.64	63,969	91	0.14	4.52 (2.27-8.98)	<0.001
	Hypertension	2,854	12	0.42	113,926	106	0.09	4.53 (2.49-8.25)	<0.001
	Chronic lung disease	1,736	9	0.52	50,850	80	0.16	3.31 (1.66-6.60)	<0.001
	None of the above	32,506	26	0.08	2,307,917	343	0.01	5.39 (3.61-8.02)	<0.001
60-69	**Total**	16,243	101	0.62	1,108,856	1,401	0.13	4.95 (4.04-6.06)	<0.001
	Diabetes	2,212	30	1.36	71,106	263	0.37	3.70 (2.53-5.42)	<0.001
	Cardiovascular disease	1,945	25	1.29	112,327	420	0.37	3.47 (2.31-5.21)	<0.001
	Hypertension	2,967	20	0.67	174,006	477	0.27	2.47 (1.58-3.87)	<0.001
	Chronic lung disease	1,289	19	1.47	42,353	296	0.70	2.13 (1.33-3.39)	0.002
	None of the above	10,899	42	0.39	847,114	616	0.07	5.32 (3.89-7.27)	<0.001

**70-79**	**Total**	9,823	139	1.42	923,261	3,852	0.42	3.43 (2.89-4.06)	<0.001
	Diabetes	1,589	39	2.45	96,742	898	0.93	2.69 (1.94-3.71)	<0.001
	Cardiovascular disease	2,132	42	1.97	190,079	1,735	0.91	2.18 (1.60-2.97)	<0.001
	Hypertension	2,685	53	1.97	256,297	1,822	0.71	2.81 (2.13-3.71)	<0.001
	Chronic lung disease	1,017	32	3.15	58,851	789	1.34	2.39 (1.67-3.42)	<0.001
	None of the above	5,428	46	0.85	563,799	1,239	0.22	3.88 (2.89-5.22)	<0.001
**80+**	**Total**	3,430	141	4.11	509,240	10,985	2.16	1.94 (1.64-2.30)	<0.001
	Diabetes	482	35	7.26	63,657	1,900	2.98	2.55 (1.80-3.60)	<0.001
	Cardiovascular disease	1,148	60	5.23	187,851	5,981	3.18	1.68 (1.29-2.18)	<0.001
	Hypertension	1,259	44	3.49	214,834	5,676	2.64	1.33 (0.99-1.80)	0.061
	Chronic lung disease	304	19	6.25	40,185	1,600	3.98	1.61 (1.01-2.56)	0.046
	None of the above	1,540	51	3.31	220,535	2,996	1.36	2.49 (1.88-3.30)	<0.001

OR, odds ratio; CI, confidence interval.

[a] Comorbidities recorded between 2013 and 2017.

[b] n Deaths counted for each comorbidity. i.e., deaths associated with more than one comorbidity will appear several times.

[c] Diagnoses of severe mental disorders (bipolar or psychotic disorder) recorded between 1998 and 2017.

[d] Small numbers withheld due to confidentiality reasons.

Supplementary Table S2: Hospitalizations associated with influenza/pneumonia between 1 January 2018 and 31 December 2019 in patients with severe mental disorder vs reference population according to age and comorbidities.

Age Group [Years]	According to comorbidity [ab]	Population with severe mental disorder [c]			Reference Population			OR (95% CI)	P value
		Population size	n	%	Population size	n	%		
Across all Age groups	**Total**	97,034	2,495	2.57	7,683,693	94,572	1.23	2.12 (2.03-2.20)	<0.001
	Diabetes	7,467	566	7.58	315,521	16,971	5.38	1.44 (1.32-1.57)	<0.001
	Cardiovascular disease	6,780	628	9.26	560,754	37,766	6.73	1.41 (1.30-1.54)	<0.001
	Hypertension	10,048	739	7.35	769,321	40,648	5.28	1.42 (1.32-1.53)	<0.001
	Chronic lung disease	5,335	531	9.95	234,227	17,469	7.46	1.37 (1.25-1.50)	<0.001
	None of the above	77,302	1,092	1.41	6,474,647	36,002	0.56	2.56 (2.41-2.72)	<0.001
20-39	**Total**	28,725	156	0.54	2,612,883	4,676	0.18	3.05 (2.60-3.57)	<0.001
	Diabetes	544	14	2.57	23,012	185	0.80	3.26 (1.88-5.65)	<0.001
	Cardiovascular disease	147	X[d]	-	6,528	X	-	-	-
	Hypertension	283	4	1.41	10,258	115	1.12	1.26 (0.46-3.45)	0.660
	Chronic lung disease	989	20	2.02	41,988	405	0.96	2.12 (1.35-3.34)	0.002
	None of the above	26,929	123	0.46	2,535,282	3,988	0.16	2.91 (2.43-3.49)	<0.001
40-59	**Total**	38,813	577	1.49	2,529,453	10,138	0.40	3.75 (3.45-4.08)	<0.001
	Diabetes	2,640	116	4.39	61,004	1,172	1.92	2.35 (1.93-2.85)	<0.001
	Cardiovascular disease	1,408	83	5.89	63,969	1,327	2.07	2.96 (2.35-3.72)	<0.001
	Hypertension	2,854	134	4.70	113,926	1,795	1.58	3.08 (2.57-3.68)	<0.001
	Chronic lung disease	1,736	109	6.28	50,850	1,295	2.55	2.56 (2.10-3.14)	<0.001
	None of the above	32,506	301	0.93	2,307,917	6,692	0.29	3.21 (2.86-3.61)	<0.001
60-69	**Total**	16,243	721	4.44	1,108,856	13,968	1.26	3.64 (3.37-3.93)	<0.001
	Diabetes	2,212	183	8.27	71,106	2,486	3.50	2.49 (2.13-2.91)	<0.001
	Cardiovascular disease	1,945	165	8.48	112,327	3,856	3.43	2.61 (2.22-3.07)	<0.001
	Hypertension	2,967	208	7.01	174,006	4,813	2.77	2.65 (2.30-3.06)	<0.001
	Chronic lung disease	1,289	172	13.34	42,353	2,748	6.49	2.22 (1.88-2.62)	<0.001
	None of the above	10,899	309	2.84	847,114	6,415	0.76	3.82 (3.41-4.29)	<0.001
	**Total**	9,823	683	6.95	923,261	27,255	2.95	2.46 (2.27-2.66)	<0.001

	Diabetes	1,589	178	11.20	96,742	6,096	6.30	1.88 (1.60-2.20)	<0.001
	Cardiovascular disease	2,132	221	10.37	190,079	11,636	6.12	1.77 (1.54-2.04)	<0.001
**70-79**	Hypertension	2,685	243	9.05	256,297	13,020	5.08	1.86 (1.63-2.12)	<0.001
	Chronic lung disease	1,017	173	17.01	58,851	6,234	10.59	1.73 (1.47-2.04)	<0.001
	None of the above	5,428	242	4.46	563,799	8,856	1.57	2.92 (2.57-3.33)	<0.001
	**Total**	3,430	358	10.44	509,240	38,535	7.57	1.42 (1.28-1.59)	<0.001
	Diabetes	482	75	15.56	63,657	7,032	11.05	1.48 (1.16-1.90)	0.002
	Cardiovascular disease	1,148	159	13.85	187,851	20,947	11.15	1.28 (1.08-1.52)	0.004
**80+**	Hypertension	1,259	150	11.91	214,834	20,905	9.73	1.25 (1.06-1.49)	0.009
	Chronic lung disease	304	57	18.75	40,185	6,787	16.89	1.14 (0.85-1.52)	0.396
	None of the above	1,540	117	7.60	220,535	10,051	4.56	1.72 (1.42-2.08)	<0.001

n, number of hospitalizations; OR, odds ratio; CI, confidence interval.
[a] Comorbidities recorded between 2013 and 2017.
[b] n hospitalizations counted for each comorbidity. i.e., hospitalizations associated with more than one comorbidity will appear several times.
[c] Diagnoses of severe mental disorders (bipolar or psychotic disorder) recorded between 1998 and 2017.
[d] Withheld due to confidentiality reasons.

Supplementary Table S3: Deaths associated with sepsis between 1 January 2018 and 31 December 2019 in patients with severe mental disorder vs reference population according to age and comorbidities.

Age Group [Years]	According to comorbidity [ab]	Population with severe mental disorder [c]			Reference Population			OR (95% CI)	P value
		Population size	Deaths	%	Population size	Deaths	%		
**Across all Age groups**	**Total**	97,034	156	0.16	7,683,693	7,666	0.10	1.61 (1.38-1.89)	<0.001
	Diabetes	7,467	40	0.54	315,521	1,865	0.59	0.91 (0.66-1.24)	1.000
	Cardiovascular disease	6,780	63	0.93	560,754	3,924	0.70	1.33 (1.04-1.71)	0.025
	Hypertension	10,048	51	0.51	769,321	3,988	0.52	0.98 (0.74-1.29)	1.000
	Chronic lung disease	5,335	32	0.60	234,227	1,181	0.50	1.19 (0.84-1.69)	0.336
	None of the above	77,302	62	0.08	6,474,647	2,232	0.03	2.33 (1.81-3.00)	<0.001
**20-39**	**Total**	28,725	0	0.00	2,612,883	40	0.00	-	-
	Diabetes	544	X[d]	-	23,012	X	-	-	-
	Cardiovascular disease	147	X	-	6,528	X	-	-	-
	Hypertension	283	0	0.00	10,258	4	0.04	-	-
	Chronic lung disease	989	0	0.00	41,988	5	0.01	-	-
	None of the above	26,929	0	0.00	2,535,282	30	0.00	-	-
**40-59**	**Total**	38,813	22	0.06	2,529,453	360	0.01	3.98 (2.59-6.13)	<0.001
	Diabetes	2,640	9	0.34	61,004	74	0.12	2.82 (1.41-5.63)	0.003
	Cardiovascular disease	1,408	9	0.64	63,969	81	0.13	5.07 (2.54-10.12)	<0.001
	Hypertension	2,854	7	0.25	113,926	91	0.08	3.08 (1.42-6.64)	0.004
	Chronic lung disease	1,736	4	0.23	50,850	41	0.08	2.86 (1.02-8.00)	0.045
	None of the above	32,506	6	0.02	2,307,917	203	0.01	2.10 (0.93-4.73)	0.073
**60-69**	**Total**	16,243	46	0.28	1,108,856	955	0.09	3.29 (2.45-4.43)	<0.001
	Diabetes	2,212	9	0.41	71,106	242	0.34	1.20 (0.61-2.33)	0.611
	Cardiovascular disease	1,945	15	0.77	112,327	328	0.29	2.65 (1.58-4.46)	<0.001
	Hypertension	2,967	12	0.40	174,006	398	0.23	1.77 (1.00-3.15)	0.051
	Chronic lung disease	1,289	10	0.78	42,353	142	0.34	2.32 (1.22-4.42)	0.01
	None of the above	10,899	20	0.18	847,114	391	0.05	3.98 (2.54-6.24)	<0.001
	**Total**	9,823	52	0.53	923,261	2,243	0.24	2.19 (1.66-2.88)	<0.001

	Diabetes	1,589	14	0.88	96,742	675	0.70	1.27 (0.74-2.15)	0.393
	Cardiovascular disease	2,132	22	1.03	190,079	1,112	0.59	1.77 (1.16-2.71)	0.008
**70-79**	Hypertension	2,685	19	0.71	256,297	1,216	0.47	1.49 (0.95-2.36)	0.083
	Chronic lung disease	1,017	13	1.28	58,851	433	0.74	1.75 (1.00-3.04)	0.049
	None of the above	5,428	21	0.39	563,799	618	0.11	3.54 (2.29-5.47)	<0.001
	**Total**	3,430	36	1.05	509,240	4,068	0.80	1.32 (0.95-1.83)	0.101
	Diabetes	482	8	1.66	63,657	874	1.37	1.21 (0.60-2.45)	0.603
	Cardiovascular disease	1,148	17	1.48	187,851	2,403	1.28	1.16 (0.72-1.88)	0.556
**80+**	Hypertension	1,259	13	1.03	214,834	2,279	1.06	0.97 (0.56-1.68)	1.000
	Chronic lung disease	304	5	1.64	40,185	560	1.39	1.18 (0.49-2.87)	0.723
	None of the above	1,540	15	0.97	220,535	990	0.45	2.18 (1.31-3.64)	0.003

OR, odds ratio; CI, confidence interval.
[a] Comorbidities recorded between 2013 and 2017.
[b] n Deaths counted for each comorbidity. i.e., deaths associated with more than one comorbidity will appear several times.
[c] Diagnoses of severe mental disorders (bipolar or psychotic disorder) recorded between 1998 and 2017.
[d] X denotes small numbers withheld due to confidentiality reasons.

Supplementary Table S4: Hospitalizations associated with sepsis between 1 January 2018 and 31 December 2019 in patients with severe mental disorder vs reference population according to age and comorbidities.

Age Group [Years]	According to comorbidity [ab]	Population with severe mental disorder [c]			Reference Population			OR (95% CI)	P value
		Population size	*n*	%	Population size	*n*	%		
**Across all Age groups**	**Total**	97,034	742	0.76	7,683,693	31,275	0.41	1.89 (1.75-2.03)	<0.001
	Diabetes	7,467	209	2.80	315,521	7,004	2.22	1.27 (1.10-1.46)	<0.001
	Cardiovascular disease	6,780	206	3.04	560,754	12,839	2.29	1.34 (1.16-1.54)	<0.001
	Hypertension	10,048	234	2.33	769,321	14,035	1.82	1.28 (1.13-1.46)	<0.001
	Chronic lung disease	5,335	141	2.64	234,227	4,159	1.78	1.50 (1.27-1.78)	<0.001
	None of the above	77,302	310	0.40	6,474,647	11,807	0.18	2.20 (1.97-2.47)	<0.001
**20-39**	**Total**	28,725	41	0.14	2,612,883	1,379	0.05	2.71 (1.98-3.69)	<0.001
	Diabetes	544	5	0.92	23,012	75	0.33	2.84 (1.14-7.04)	0.024
	Cardiovascular disease	147	0	0.00	6,528	53	0.81	-	-
	Hypertension	283	X[d]	-	10,258	X	-	-	-
	Chronic lung disease	989	4	0.40	41,988	74	0.18	2.30 (0.84-6.30)	0.105
	None of the above	26,929	34	0.13	2,535,282	1,159	0.05	2.76 (1.96-3.89)	<0.001
**40-59**	**Total**	38,813	194	0.50	2,529,453	3,853	0.15	3.29 (2.85-3.80)	<0.001
	Diabetes	2,640	58	2.20	61,004	610	1.00	2.22 (1.69-2.92)	<0.001
	Cardiovascular disease	1,408	26	1.85	63,969	580	0.91	2.06 (1.38-3.06)	<0.001
	Hypertension	2,854	44	1.54	113,926	888	0.78	1.99 (1.47-2.70)	<0.001
	Chronic lung disease	1,736	28	1.61	50,850	331	0.65	2.50 (1.70-3.69)	<0.001
	None of the above	32,506	92	0.28	2,307,917	2,411	0.10	2.71 (2.20-3.34)	<0.001
**60-69**	**Total**	16,243	215	1.32	1,108,856	5,513	0.50	2.68 (2.34-3.08)	<0.001
	Diabetes	2,212	60	2.71	71,106	1,263	1.78	1.54 (1.19-2.00)	0.0013
	Cardiovascular disease	1,945	59	3.03	112,327	1,716	1.53	2.02 (1.55-2.62)	<0.001
	Hypertension	2,967	61	2.06	174,006	2,216	1.27	1.63 (1.26-2.10)	<0.001
	Chronic lung disease	1,289	47	3.65	42,353	697	1.65	2.26 (1.67-3.06)	<0.001
	None of the above	10,899	87	0.80	847,114	2,408	0.28	2.82 (2.28-3.50)	<0.001
	**Total**	9,823	208	2.12	923,261	9,763	1.06	2.02 (1.76-2.33)	<0.001
	Diabetes	1,589	61	3.84	96,742	2,673	2.76	1.40 (1.08-1.82)	0.010

70-79	Cardiovascular disease	2,132	83	3.89	190,079	4,400	2.31	1.71 (1.37-2.13)	<0.001
	Hypertension	2,685	92	3.43	256,297	4,964	1.94	1.80 (1.46-2.22)	<0.001
	Chronic lung disease	1,017	49	4.82	58,851	1,574	2.67	1.84 (1.38-2.47)	<0.001
	None of the above	5,428	67	1.23	563,799	3,098	0.55	2.26 (1.77-2.89)	<0.001
80+	**Total**	3,430	84	2.45	509,240	10,767	2.11	1.16 (0.94-1.44)	0.176
	Diabetes	482	25	5.19	63,657	2,383	3.74	1.41 (0.94-2.11)	0.098
	Cardiovascular disease	1,148	38	3.31	187,851	6,090	3.24	1.02 (0.74-1.41)	0.905
	Hypertension	1,259	37	2.94	214,834	5,967	2.78	1.06 (0.76-1.47)	0.741
	Chronic lung disease	304	13	4.28	40,185	1,483	3.69	1.17 (0.67-2.04)	0.602
	None of the above	1,540	30	1.95	220,535	2,731	1.24	1.58 (1.10-2.28)	0.013

n, number of hospitalizations. OR, odds ratio; CI, confidence interval.

[a] Comorbidities recorded between 2013 and 2017.

[b] n Deaths counted for each comorbidity. i.e., deaths associated with more than one comorbidity will appear several times.

[c] Diagnoses of severe mental disorders (bipolar or psychotic disorder) recorded between 1998 and 2017.

[d] X denotes small numbers withheld due to confidentiality reasons.

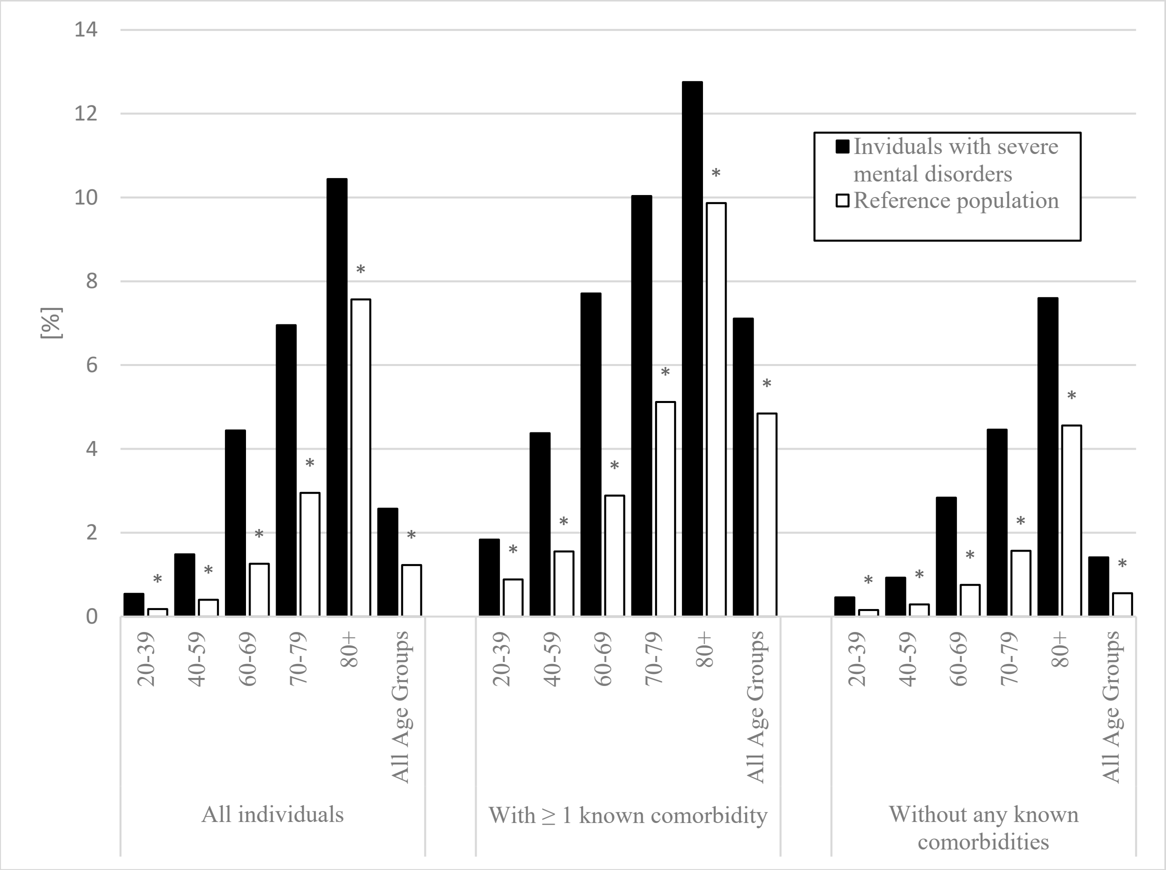

Supplementary Figure S1. Hospitalizations associated with influenza/pneumonia between 2018-2019 in individuals with severe mental disorders and the general population. Hospitalizations presented as a percentage of population size. Diagnosis of severe mental disorder (bipolar or psychotic disorder) recorded between 1998 and 2017. Comorbidities defined as diabetes, hypertension, cardiovascular disease and/or chronic lung disease recorded between 2013 and 2017. Significant differences are highlighted with an asterisk (*: p <0.05).

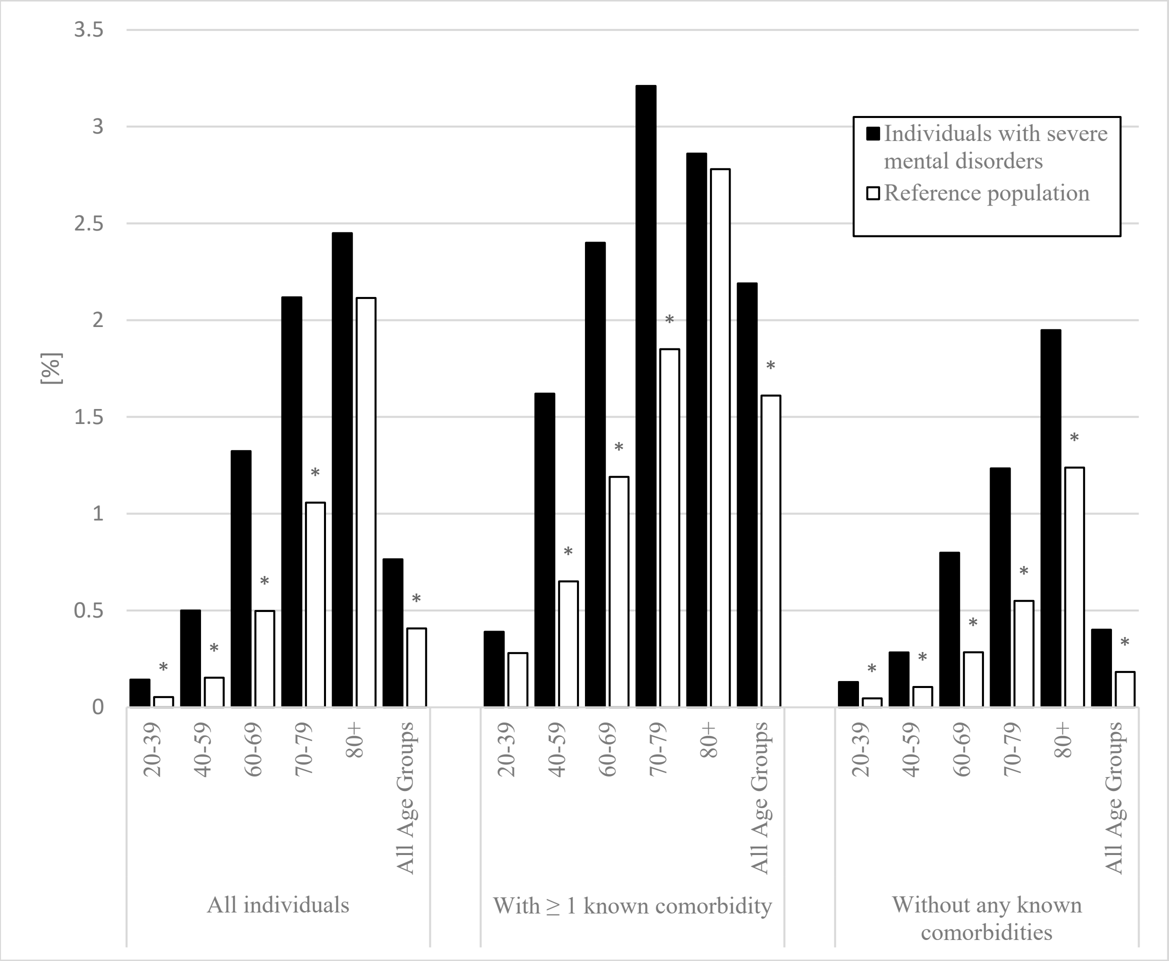

Supplementary Figure S2. Hospitalizations associated with sepsis between 2018-2019 in individuals with severe mental disorders and the general population. Hospitalizations presented as a percentage of population size. Diagnosis of severe mental disorder (bipolar or psychotic disorder) recorded between 1998 and 2017. Comorbidities defined as diabetes, hypertension, cardiovascular disease and/or chronic lung disease recorded between 2013 and 2017. Significant differences are highlighted with an asterisk (*: $p < 0.05$).
